# A Multipollutant Approach to Estimating Causal Effects of Air Pollution Mixtures on Overall Mortality in a Large, Prospective Cohort

**DOI:** 10.1097/EDE.0000000000001492

**Published:** 2022-04-05

**Authors:** Eugenio Traini, Anke Huss, Lützen Portengen, Matti Rookus, W. M. Monique Verschuren, Roel C. H. Vermeulen, Andrea Bellavia

**Affiliations:** From the a Institute for Risk Assessment Sciences, Utrecht University, Utrecht; b Department of Epidemiology, Netherlands Cancer Institute (NKI), Amsterdam; c Centre for Nutrition, Prevention and Health Services, National Institute for Public Health and the Environment, Bilthoven; d Julius Center for Health Sciences and Primary Care, University Medical Center Utrecht, Utrecht University, Utrecht, the Netherlands; e Department of Environmental Health, Harvard T.H. Chan School of Public Health, Boston, MA.

**Keywords:** Air pollution, Mortality, Mixture, Interaction, Machine learning, Causal methods, Propensity score

## Abstract

Several studies have confirmed associations between air pollution and overall mortality, but it is unclear to what extent these associations reflect causal relationships. Moreover, few studies to our knowledge have accounted for complex mixtures of air pollution. In this study, we evaluate the causal effects of a mixture of air pollutants on overall mortality in a large, prospective cohort of Dutch individuals.

**Methods:**

We evaluated 86,882 individuals from the LIFEWORK study, assessing overall mortality between 2013 and 2017 through national registry linkage. We predicted outdoor concentration of five air pollutants (PM_2.5_, PM_10_, NO_2_, PM_2.5_ absorbance, and oxidative potential) with land-use regression. We used logistic regression and mixture modeling (weighted quantile sum and boosted regression tree models) to identify potential confounders, assess pollutants' relevance in the mixture–outcome association, and investigate interactions and nonlinearities. Based on these results, we built a multivariate generalized propensity score model to estimate the causal effects of pollutant mixtures.

**Results:**

Regression model results were influenced by multicollinearity. Weighted quantile sum and boosted regression tree models indicated that all components contributed to a positive linear association with the outcome, with PM_2.5_ being the most relevant contributor. In the multivariate propensity score model, PM_2.5_ (OR=1.18, 95% CI: 1.08–1.29) and PM_10_ (OR=1.02, 95% CI: 0.91–1.14) were associated with increased odds of mortality per interquartile range increase.

**Conclusion:**

Using novel methods for causal inference and mixture modeling in a large prospective cohort, this study strengthened the causal interpretation of air pollution effects on overall mortality, emphasizing the primary role of PM_2.5_ within the pollutant mixture.

Exposure to air pollution has been found to be associated with higher mortality rates in several studies over the last decades,^1–3^ and associations have been reported even at low levels of exposure.^2,4–7^ However, to improve our understanding of these associations and to facilitate the development of better targeted public health regulations and interventions, it is important to determine to which extent these associations reflect causal relationships.^8^

When evaluating the health effects of environmental exposures such as air pollutants, it is important to account for the co-occurrence of multiple environmental constituents, present in the real world as a complex mixture.^9^ To evaluate the causal effects of air pollution on health, it is thus critical that studies account for this complex nature of exposure. This approach would allow identifying relevant contributors within the mixture as well as detecting potential interactions between pollutants. Several analytical methods have been proposed to deal with statistical challenges inherent to mixtures, such as co-exposure confounding, high correlation, and interaction between components of the mixture.^10–12^ Furthermore, regulatory policies are still mostly designed to regulate one pollutant or one source at a time, whereas more complex evaluations regarding causality may possibly lead to a more targeted regulatory policy.^8^ As such, there is a need to improve our understanding of the causal effects of environmental mixtures evaluated as a complex exposure situation of high-dimensional data.^13,14^

In this study, we investigated the effects of a mixture of five pollutants on overall mortality in a large population-based cohort of Dutch individuals where air pollution exposure has been assessed through state-of-the-art methodologies. We adopted a pluralistic approach exploring the pollutant mixture with targeted methods for high-dimensional exposures, including boosted regression tree and weighted quantile sum models and investigated the causal relationships between multiple pollutants and mortality with novel extensions of propensity score approaches.

## MATERIAL AND METHODS

### Study Participants and Outcome Definition

We used data from the LIFEWORK study, a large prospective cohort consortium comprising nearly 90,000 participants aged 18+ living in the Netherlands. LIFEWORK was designed as a federated study resulting from the integration of three existing Dutch cohorts: the Nightingale study, initiated in 2011 and the largest contributor to the LIFEWORK study (68%), the Occupational and Environmental Health Cohort Study (AMIGO) (17%) started in 2011, and the European Prospective Investigation into Cancer and Nutrition in the Netherlands (EPIC-NL) (15%), established between 1993 and 1997. Data were collected from each cohort between 2011 and 2012 (baseline questionnaires for AMIGO and Nightingale, follow-up questionnaire for EPIC-NL) and pooled to set up the LIFEWORK cohort, setting the baseline at January 1, 2013. The rationale, study design, and participant recruitment in LIFEWORK were discussed in detail elsewhere.^15–18^ The contributing subcohorts were approved by the local research ethics review committee or institutional review board (AMIGO and EPIC-NL Prospect by the committee at the University Medical Center Utrecht; EPIC-NL MORGEN by the committee at TNO Nutrition and Food Research; and Nightingale by the committee at the Netherlands Cancer Institute), and participants signed an informed consent form for each subcohort prior to enrolment.

From the original 88,466 LIFEWORK participants, we excluded 683 individuals with missing exposure information (their residential address either was incomplete; fell in the sea, river, or another watercourse; or at least one predictor for the land-use regression models was missing), 378 with reported emigration during the study, and 523 with no informed consent to link to the Municipal Personal Records Database (GBA). The GBA is a centralized automated population registration system that holds information on residence (home address) and date of death of people who reside in the Netherlands as well as personal data on migration. After exclusions, the total population evaluated in this study consisted of 86,882 individuals.

The outcome of interest was all-cause mortality, assessed by ascertaining vital status from the Dutch Central Bureau of Statistics (CBS) and date of death over a 5-year follow-up period (1 January 2013 to 31 December 2017) via data linkage to the GBA.

### Exposure Assessment

We evaluated air pollution as a mixture of five components: particulate matter with aerodynamic diameter less than 2.5 μm (PM_2.5_), particulate matter with aerodynamic diameter less than 10 μm (PM_10_), a marker of diesel exhaust particulate (PM_2.5_ absorbance), nitrogen dioxide (NO_2_), and the oxidative potential estimated in PM_2.5_ by dithiothreitol.

Land-use regression models were fitted to estimate outdoor concentrations of air pollutants at the home address for each participant, combining monitoring of air pollution at different locations and predictor variables obtained from spatial data.^19^ Model development has been described in detail elsewhere.^6^ Briefly, we developed land-use regression models based upon annual average concentrations of PM_2.5_, PM_2.5_ absorbance, PM_10_ and NO_2_ measured between October 2008 and April 2011 during three 14-day periods to account for seasonal variation. We conducted measurements in 20 European study areas at 20–40 sites for PM and at 40–80 sites per area for NO_2_. The annual average ambient pollutant concentrations were estimated at addresses of study participants at baseline using as predictor variables data on traffic intensity, household density, land use, and other study-area variables such as altitude and distance to the sea. The median model explained variance (R^2^) ranged from 71% (PM_2.5_) to 89% (PM_2.5_ absorbance).^5,20^ Oxidative potential concentration was estimated based on a sampling period of three 2-week PM measurements carried out at 40 sites spread over the Netherlands and Belgium between February 2009 and February 2010 taking into account temporal variability. Land-use regression models for oxidative potential were estimated at participants’ addresses at baseline and achieved an *R*^2^ value of 60%.^21^

### Covariates

We selected potential confounders of the associations between air pollution and overall mortality *a priori* based on results from preliminary studies.^5,6,20^ These potential confounders included age, sex, body mass index [BMI, weight (kg)/height (m)^2^], cardiovascular disease (CVD) diagnosis, chronic obstructive pulmonary disease (COPD) diagnosis, cancer diagnosis, smoking status (never, former, current), highest level of education attained (low, intermediate, high), the estimated monthly household income of the neighborhood based on income data provided by CBS in 2012 (www.cbs.nl), and the normalized difference vegetation index which quantifies vegetation density around each participant’s address based on Landsat 8 satellite images taken in 2008.^22^

### Statistical Analysis

Descriptive statistics of the study population were evaluated overall and by levels of air pollution exposure. As the interest of this analysis was in pollutant mixtures, we identified profiles of pollutant mixture exposure through K-means cluster analysis. We evaluated the correlation between pollution components by calculating Spearman’s rank correlation coefficients.

We first evaluated the association between air pollution constituents and overall mortality with classical regression models, both independently (one model for each mixture component) as well as mutually adjusting pollutants in the same statistical model. In the primary analysis, mutual adjustment was performed by considering the full set of components available in the LIFEWORK cohort. Overall mortality was evaluated as a binary outcome (dead/alive) with logistic regression, estimating ORs for mortality risk, as well as with Poisson and Cox models to account for the duration of follow-up and for possible changes in event rates over time. A sensitivity analysis was conducted using multiple imputation by chained equation (MICE) to impute missing values in the exposures.^23^ Age, sex, BMI, smoking, and CVD diagnosis were specified as predictors in the algorithm for each incomplete exposure variable. An additional sensitivity analysis was performed by excluding individuals with baseline CVD diagnosis (angina, heart attack, transient ischemic attack, stroke, other heart conditions, defined according to ICD-9 and ICD-10), COPD, and cancer diagnosis. Last, we conducted a secondary analysis on overall mortality and a subset of components (NO_2_, PM_2.5_, PM_10_) representing a group of already regulated pollutants based on existing legislation.^1^

We used multiple regression models to identify confounders of the association to be evaluated in causal models. Specifically, we first evaluated a fully adjusted multiple regression model by adjusting for all covariates presented in the previous section and then removed those confounders that did not change any exposure coefficient by more than 10%. To assess the impact of multicollinearity of multiple regression estimates, we calculated variance inflation factors (VIFs).

To address issues of multicollinearity and to identify pollution constituents from clusters of correlated exposures that should be included in the causal analysis, we used weighted quantile sum and boosted regression tree models. In brief, these methods are techniques used in mixture modeling to identify the relative contribution of several exposures in the overall effect between the mixture and the outcome of interest, while accounting for high correlation structures.^24,25^ While both correlation analysis and multivariable regression can inform on the levels of correlation, neither of them can detect which covariates within the mixture are driving the associations, and to what extent. A weighted quantile sum summarizes the mixtures with a single index estimated as a weighted linear combination of the exposures and allows identifying the relative contribution of each mixture constituent. This technique makes the assumptions of linear associations on the quantile scale and of unidirectionality (all exposures-outcome associations are either positive or negative), but directly provides an estimate of the relative percent contribution of each exposure within the mixture.^24^ Boosted regression tree, on the other hand, is a machine learning technique based on tree modeling that does not provide any estimate of exposures contribution but allows ranking their relative importance while relaxing assumptions of unidirectionality and linearity, strengthening the interpretation of the results from the weighted quantile sum. In addition, boosted regression tree provides a qualitative assessment of interactions' importance (through the use of the measure called H-statistics), which can be used as an exploratory tool to detect two-way or higher-order interactions that should be incorporated in subsequent analyses.^25,26^

To estimate the causal effects of pollutant mixture on overall mortality we used propensity score methods, building the propensity scores from the set of confounders identified in the regression modeling.^27^ Propensity score methods achieve balance across a set of confounders thus reducing the confounding effect in the exposure–outcome relation. To evaluate pollutants as continuous exposures, we used the generalized propensity scores extension, which handles single continuous exposures given a set of confounders,^28,29^ under the assumption that exposures follow a normal distribution. We first used generalized propensity scores to generate weights for each continuous exposure separately.^30^ Next, to account for the mixture nature of air pollution, we used the multivariate generalized propensity score, a novel extension of the generalized propensity score for multiple simultaneous continuous exposures implemented in the R package *mvGPS*.^31^ Multivariate generalized propensity score has the advantage over generalized propensity score of simultaneously estimating weights for multivariate continuous exposures that are constructed as the ratio of the marginal density of the exposures to the conditional density.^31^ Specifically, the multivariate generalized propensity score generates stabilized inverse probability of treatment weights (IPTWs) assuming a multivariate normal distribution for the simultaneous exposures. These weights have been shown to balance confounders and provide unbiased exposure–response estimates.^32^ To optimize propensity score weights and avoid possible effects due to extreme weights, the procedure allows trimming both the upper and lower bounds of the weights’ distribution.^33^ We conducted the main analysis using the recommended weights threshold at the 99th percentile,^31^ and evaluated other thresholds (0.97, 0.95) in sensitivity analyses. All analyses were conducted with the R statistical software, version 4.0.4. Computing code related to all analyses presented is publicly available at https://github.com/andreabellavia/causalpm, also presenting different approaches to deal with categorical confounders, option that is not automatized in the current version of the *mvGPS* package (1.2.1) and requires additional coding. All exposures were evaluated as continuous variables and results indicate changes per interquartile range width (IQRw) increase in mean air pollution exposure.

## Results

Baseline characteristics of the study population, overall and by levels of air pollution exposures, are presented in Table 1. K-means clustering identified three groups as the optimal characterization of the mixture, with the clusters summarizing levels of low, moderate, and high exposure to air pollution. Individuals with higher levels of exposure were on average older, lived in areas with lower normalized difference vegetation index, and were more likely to be smokers. Figure presents the correlation structure between air pollution constituents at baseline, while eTable 1; http://links.lww.com/EDE/B920 provides the distribution of each pollutant at baseline. All mixture components were highly positively correlated with each other.

**TABLE 1.** Baseline Characteristics of the LIFEWORK Participants and Estimated Annual Pollutant Exposures^a^ at Subject Recruitment, Overall and by Levels of Pollution Exposure^b^

	Low Exposure	Moderate Exposure	High Exposure	Overall
	(*N*=34,018)	(*N*=37,853)	(*N*=15,011)	(*N*=86,882)
No. of participants (%)				
Amigo	22	15	10	17
EPIC	9	16	28	15
Nightingale	69	69	62	68
Age (years)				
Mean (SD)	48.8 (11.6)	50.5 (12.9)	52.2 (14.3)	50.2 (12.7)
Sex (%)				
Male	12	9	9	11
Female	88	91	91	89
Highest level of education attained^c^ (%)				
Low	11	14	18	14
Intermediate	48	43	35	44
High	41	43	47	42
Missing	0.2	0.2	0.3	0.2
Smoking status (%)				
Never	48	47	44	46
Former	40	40	40	40
Current	11	12	14	13
Missing	0.6	1.0	1.7	1.0
Body mass index (kg/m^2^)				
Mean (SD)	25.2 (4.16)	25.3 (4.30)	25.2 (4.41)	25.3 (4.26)
Missing (%)	0.4	0.6	0.9	0.6
CVD diagnosis at baseline (%)				
Negative	93	93	90	92
Positive	7	7	10	8
COPD diagnosis at baseline (%)				
Negative	98	97	96	97
Positive	2	3	4	3
Cancer diagnosis at baseline (%)				
Negative	98	97	95	97
Positive	2	3	5	3
Monthly income estimate^d^				
Mean (SD)	2,590 (764)	2,800 (890)	2,870 (1,000)	2,730 (873)
Missing (%)	5.1	3.4	3.3	4.0
Normalized difference vegetation index				
Mean (SD)	0.571 (0.0844)	0.503 (0.0796)	0.448 (0.0864)	0.520 (0.0943)
Missing (%)	2.6	1.2	0.9	1.7
NO_2_ (μg/m^3^)				
Mean (SD)	17.7 (2.55)	24.9 (2.25)	33.7 (4.30)	23.6 (6.34)
PM_2.5_ (μg/m^3^)				
Mean (SD)	16.3 (0.721)	16.7 (0.559)	17.0 (0.707)	16.6 (0.704)
PM_2.5_ absorbance (10^−5^ m^−1^)				
Mean (SD)	1.09 (0.132)	1.29 (0.125)	1.57 (0.227)	1.26 (0.225)
PM_10_ (μg/m^3^)				
Mean (SD)	24.1 (0.381)	24.7 (0.643)	26.4 (1.42)	24.8 (1.12)
Oxidative potential (nmol DTT/min/m^3^)				
Mean (SD)	1.06 (0.208)	1.22 (0.165)	1.32 (0.119)	1.17 (0.202)

a Air pollution levels were estimated at baseline based on annual average concentrations measured between October 2008 and April 2011 (NO_2_, PM_2.5_, PM_2.5_ absorbance, PM_10_) and between February 2009 and February 2010 (Oxidative Potential).

b Low, medium, and high levels of exposures derived with cluster analysis.

c Low: primary school, lower vocational training or lower secondary education; intermediate: intermediate vocational education or intermediate/higher secondary education; high: higher vocational education or university degree.

d Household income was estimated based on participants’ baseline postal code. Each postal code was linked to income data from Statistics Netherlands for December 2012.

CVD, cardiovascular disease; COPD, chronic obstructive pulmonary disease; SD, standard deviation.

**FIGURE.**
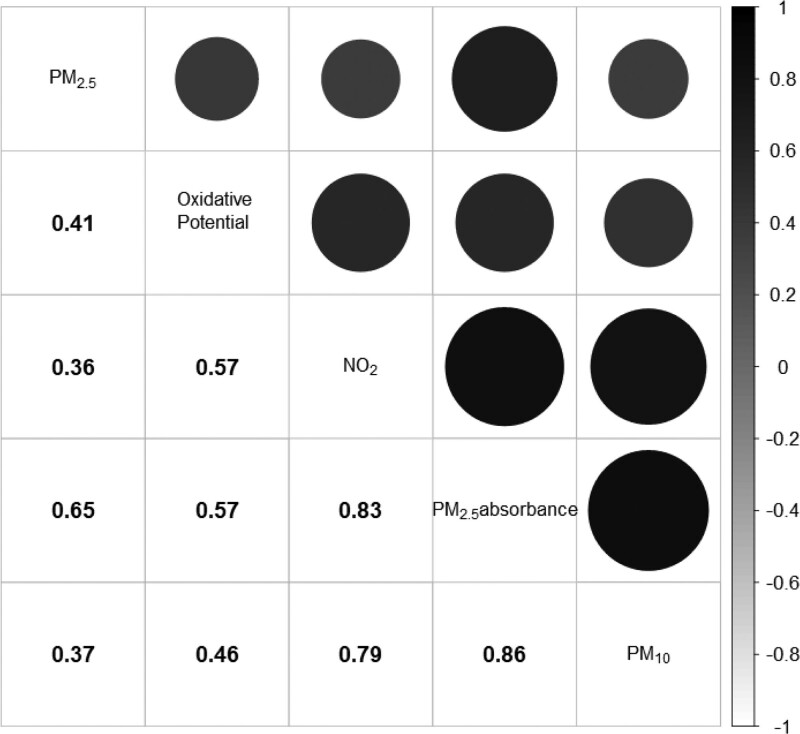
Spearman rank correlation coefficients and correlation plot of air pollution constituents at baseline (2008–2011). Darker colors and larger circles indicate higher positive correlation levels.

During 5 years of follow-up, we observed 1071 deaths (1.2%). Results from logistic regression models are reported in Table 2 and eTable 2; http://links.lww.com/EDE/B920. Out of all potential confounders evaluated in fully adjusted models, only age, sex, BMI, smoking, and baseline CVD diagnosis met the criteria for confounding to be selected for inclusion in the final model (referred to, in tables, as minimally adjusted model). When mutually adjusting the full set of air pollution constituents in the same statistical model, both PM_2.5_ and PM_10_ were associated with higher odds of mortality (respectively, OR=1.17, 95% CI: 0.99–1.37; OR=1.21, 95% CI: 1.03–1.42), even though VIFs for these coefficients were relatively high (Table 2). PM_2.5_ absorbance was associated with a reduction in the odds of mortality, but the extremely high VIF associated with this coefficient suggests that this result might be due to (multi)collinearity. Results from the multivariable logistic regression model using MICE to impute the missing exposures showed no discrepancies from findings on complete cases (eTable 3; http://links.lww.com/EDE/B920). When mutually adjusting the models for a subset of air pollution constituents represented by NO_2_, PM_2.5_, and PM_10_, both PM_2.5_ (OR=1.03, 95% CI: 0.94–1.14) and PM_10_ (OR=1.06, 95% CI: 0.95–1.17) showed a positive, albeit much weaker, association with overall mortality (eTable 4; http://links.lww.com/EDE/B920). We observed negligible differences when excluding individuals with baseline CVD, and when using Poisson (data not shown) or Cox models (eTable 5; http://links.lww.com/EDE/B920). We, therefore, chose to only present results from logistic regression, as this allows a direct comparison with the statistical methods we used in our study to explore causal relationships, for which time-to-event models are not currently available.

**TABLE 2.** Odds Ratios of Overall Mortality Per Interquartile Range Width Increase in Mean Air Pollution Exposure, Evaluated With a Multivariable Logistic Regression Model

	Multivariable Model With Minimal Adjustment^a^		
Constituent	OR	95% CI	VIF
NO_2_	0.98	(0.82–1.18)	5.11
PM_2.5_	1.17	(0.99–1.37)	4.03
PM_2.5_ absorbance	0.74	(0.55–0.98)	18.60
PM_10_	1.21	(1.03–1.42)	7.22
Oxidative potential	1.07	(0.96–1.19)	1.58

a Age, sex, BMI, smoking, CVD diagnosis.

BMI, body mass index; CVD, cardiovascular disease; OR, odds ratio; CI, confidence interval; VIF, variance inflation factor.

To evaluate the mixture of pollutants while accounting for the strong correlations, we estimated the relative contribution of each exposure in the mixture–outcome association with boosted regression tree and weighted quantile sum models. In the boosted regression tree model, which provides a nonparametric estimation that accounts for nonlinearities and interactions, all measures of H-statistics were consistently low, indicating a negligible impact of interactions in the mixture–outcome association (eFigure 1; http://links.lww.com/EDE/B920), and confirmed that exposure–response relationships were mostly linear and positive or null for all mixture components (data not shown). As such, weighted quantile sum assumptions were met, and this method could be used to provide an accurate estimate of the relative importance of the mixture components. Estimates of weighted quantile sum weights, presented in eFigure 2; http://links.lww.com/EDE/B920, show a prominent role of PM_2.5_ in the association, greatly surpassing the contribution of PM_10_ and other components of the mixture. Moreover, the negligible weight associated with PM_2.5_ absorbance indicates that the negative association observed in multiple regression for that variable is likely due to (multi)collinearity. The association between the overall mixture and mortality, estimated by the weighted quantile sum index, was negligible in our population (β=0.01, 95% CI: −0.03 to 0.04) (eFigure 3; http://links.lww.com/EDE/B920).

Based on results from multiple regression and mixture modeling, we built propensity score models using the minimal set of confounders (age, sex, BMI, smoking, CVD diagnosis), and all exposures were included in the models as continuous covariates, thus evaluating their linear effect on the outcome. Furthermore, based on results from boosted regression tree and weighted quantile sum models, we excluded PM_2.5_ absorbance from the analysis to limit the impact of multicollinearity on the results.

Table 3 presents results from the univariate and multivariate generalized propensity score models, with the recommended weights trimming at 0.99. All exposures met the normality distribution assumption required by these techniques. PM_2.5_ was associated with increased odds of mortality (OR=1.18, 95% CI: 1.08–1.29). PM_10_ was also associated with increased odds of mortality, even though the coefficient was attenuated (OR=1.02, 95% CI: 0.91–1.14) as compared to those from the multiple regression model. Results that considered alternative trimming are shown in eTable 6 (http://links.lww.com/EDE/B920) and indicate no discrepancies with the main finding.

**TABLE 3.** Odds Ratios of Overall Mortality Per Interquartile Range Width Increase in Mean Air Pollution Exposure, Evaluated With Univariate and Multivariate Generalized Propensity Score^a^ Models^b^

	GPS		mvGPS	
Constituent	OR	95% CI	OR	95% CI
NO_2_	1.10	(1.01–1.19)	1.13	(0.97–1.31)
PM_2.5_	1.11	(1.03–1.20)	1.18	(1.08–1.29)
PM_10_	1.08	(1.02–1.15)	1.02	(0.91–1.14)
Oxidative potential	1.09	(1.00–1.19)	0.97	(0.89–1.06)

a Trimming 0.99.

b PS based on age, sex, BMI, smoking, CVD diagnosis.

BMI, body mass index; CVD, cardiovascular disease; OR, odds ratio; CI, confidence interval; GPS, generalized propensity score; mvGPS, multivariate generalized propensity score.

## DISCUSSION

In this study, conducted on a large sample of individuals from the Dutch general population, we observed positive associations between air pollution mixtures and all-cause mortality, with PM_2.5_ being the main driver of the associations. Through the application of causal modeling approaches for environmental mixtures, we strengthened the causal interpretation of these findings, observing a strong effect of PM_2.5_ and a moderate effect of PM_10_.

Our findings are in line with results from previous studies,^7,34,35^ with the Netherlands being characterized by homogeneous geographic conditions due to its relatively small land extension and high population density compared to other geographic areas around the globe. In this regard, a recent systematic review supporting the derivation of updated guidelines by the World Health Organization (WHO) on PM exposure and mortality, highlighted the importance of considering the heterogeneity of study location and population characteristics, as well as level and composition of PM, among others, when interpreting and comparing results from different studies.^36^

The potential harmful effects of air pollution on overall mortality have been the primary focus of extensive research over the last decades.^1–4^ Associations have been repeatedly observed all over the world, and recent studies have also suggested that associations might follow linear relationships where even low levels of pollution might be harmful to health.^2,7,37^ Nevertheless, several research gaps in air pollution epidemiology remain to be addressed. First, air pollution is a complex exposure that should be characterized as a mixture, with different components and constituents possibly operating through either similar or different biologic pathways in the human body.^38–43^ Extensive work has been devoted to the development of high-resolution concentration surfaces of the different components and constituents of the complex ambient air pollution exposure.^24–31^ Epidemiologic studies, however, are mostly evaluating air pollution components one by one and switching the focus to air pollution as an environmental mixture has been advocated.^44^ Second, to improve our understanding of the mechanisms through which air pollution operates and to allow the development of more stringent public health regulations and interventions, it is important to determine to which extent these associations reflect causal relationships.^8^ Methods to address causality in observational studies are widely available,^45,46^ and several reports have discussed the application of these techniques in air pollution epidemiology.^13,14^ It is also desirable that such causal modeling approaches will account for the complex nature of air pollution as a mixture.^13,14^

To the best of our knowledge, this study was one of the first attempts to assess the causal effects of a mixture of air pollutants in a large population-based study. Our results confirm previous findings observed in this and other cohorts, showing a positive linear association between pollution components such as PM_2.5_ and PM_10_ and overall mortality. In addition, by jointly evaluating several components in the same statistical framework, we observed that PM_2.5_ seems to be the strongest predictor of overall mortality and that interactive mechanisms were not influential in our cohort. The possible mechanisms through which PM_2.5_ operates are increased systemic inflammation and oxidative stress, increased blood pressure, and reduced lung function, thus resulting in a greater risk of cardiovascular and respiratory morbidity.^37^ Results are consistent across the different methods applied, with the largest effect on overall mortality obtained for PM_2.5_ using the multivariate generalized propensity score. This method possibly provides, on theoretical grounds, more robust estimates compared to both the univariable and multivariable logistic regression, and the univariate generalized propensity score. However, due to the lack of studies that have previously applied this extension of the propensity score in epidemiologic settings, and therefore the inability to directly compare our findings with those obtained in other cohorts, this result must be interpreted with caution. The 2019 Integrated Science Assessment (ISA) released by US Environmental Protection Agency (EPA) rated the association between PM_2.5_ and natural-cause mortality as suggestive,^47^ contrary to PM_10_ which was already fully recognized as harmful to human health. Our results, by distinguishing the roles of PM_10_ and PM_2.5_, and showing the prominent role of the latter in our study population, provide relevant results that can inform future public health policies.

This study has several strengths. First, it is one of the first studies to evaluate the causal effects of air pollution while jointly evaluating several pollutants components as an environmental mixture. Specifically, we used a recent extension of the generalized propensity score, the multivariate generalized propensity score approach, that, to our knowledge, has never been used before in environmental epidemiology. While making the assumption that all evaluated exposures are normally distributed, the multivariate score improves on several aspects as compared with other approaches. First, the propensity score is a balancing score, which means that conditioning on propensity score via regression adjustment implies that individuals within the same strata of the propensity score should be identical in terms of their observable characteristics, regardless of their level of treatment.^28,29^ Thanks to the balancing property, the propensity score thus removes sources of potential confounding and returns valid estimates by balancing covariates to predict the probability of exposure.^27^ Second, the multivariate generalized propensity score approach has the ability of simultaneously estimating propensity score weights for each exposure, thus achieving superior balance compared to univariate alternatives. In addition, through the multivariate score, it is possible to specify multiple sets of confounders for each exposure of interest reflecting many real-world settings in which the confounders may actually differ across exposure variables. Finally, the option to trim extreme weights at a particular percentile and the wide number of metrics that can be used to select and compare different propensity score approaches, make the multivariate generalized propensity score a method well suited to get more robust estimates on the joint effect of multiple continuous exposures on health outcomes, confirming and possibly strengthening results obtained with more traditional methods. We recommend that future studies validate our results in other cohorts with this or alternative causal modeling techniques. Second, we used a pluralistic approach integrating several statistical methods for causal inference and environmental mixtures.^46^ To identify relevant predictors within the air pollution mixture we used two statistical methods, namely weighted quantile sum and boosted regression tree, that allow ranking the importance of exposures in the overall mixture–outcome association, thus informing which regression results might be biased due to the high correlation. In this study, multiple regression results were influenced by (multi)collinearity due to the high correlation structure, particularly PM_2.5_ absorbance which was shown to be mostly irrelevant in the mixture–outcome association once the high correlation was accounted for. Third, we used data from a large population of Dutch individuals with a prospective design, and a high-resolution assessment of air pollution components, all elements that further enhance the robustness of our results and the causal interpretation of these findings.

A limitation of this study is the relatively short duration of follow-up that did not allow us to thoroughly evaluate how the effects of air pollution may change over time. Future studies with longer follow-up should replicate these analyses and evaluate overall mortality as a time-to-event outcome for those statistical techniques where this extension is available. Moreover, no information was available on air pollution levels other than those modeled at the participants’ home addresses, thus precluding the possibility to quantify the exposure in places where participants could have spent some of their time during the day or when moving from one place to another. Furthermore, information on emigration time was not available for the majority of participants who had emigrated during the follow-up. As such, these individuals had to be excluded from the analysis. In addition, despite several sociodemographic covariates that were available and could be investigated as potential confounders of the associations, we cannot exclude the presence of residual confounding due to variables that were not available in this study. Exposures were derived using land-use regression models, which might introduce more complexity due to the use of shared predictors that may lead to stronger correlations between exposures than those existing in the real world.^48^ In large cohorts, such as the one we considered in our study, it is usually difficult or impossible to directly measure the different pollutants for each participant due to logistics complexity and the high costs associated, and therefore it is common to rely on exposure modeling. This is also suggested by WHO which indicates that exposure modeling is a logical or empirical construct that allows the estimation of an individual or population exposure parameters from available input data.^49^ Finally, in this first attempt to evaluate the causal effects of air pollution mixture we only focused on five major components of air pollution that had been assessed in this cohort. Future studies within LIFEWORK should consider finer pollution characterization, once this is available, by integrating additional components into the models, such as ultrafine particles, black carbon, as well as PM elemental constituents. Also, future studies could further expand analyses to include additional environmental risk factors (water pollution, noise, electromagnetic fields) and relevant conditions, such as lung cancer or respiratory diseases, making use of the statistical methods we proposed in our study to account for complex interrelations between risk factors in real-life settings. These results should also advise quantitative researchers to study and develop novel methods that could improve our understanding of the causal effects of complex mixtures of environmental pollutants.

In conclusion, this study strengthened the causal interpretation of air pollution effects on mortality while also accounting for the complex nature of the exposure as an environmental mixture. We encourage air pollution researchers to further study the causal effects of air pollution mixtures to continue improving our scientific knowledge on the relationship between air pollution and health outcomes, and to facilitate governmental bodies to better target regulations thanks to the identification of the strongest contributor(s) to overall mortality from a complex mixture.

## ACKNOWLEDGMENTS

The authors are greatly indebted to all LIFEWORK participants. They are also grateful to Inka Pieterson at IRAS for the data management.

## Supplementary Material


