# Supplementary material for: A Multipollutant Approach to Estimating Causal Effects of Air Pollution Mixtures on Overall Mortality in a Large, Prospective Cohort
Source: Epidemiology. 2022 Apr 5;33(4):514–22. doi: 10.1097/EDE.0000000000001492 (PMC9148665; doi:10.1097/EDE.0000000000001492)

eTable 1. Distribution of the estimated annual pollutant exposures at baseline in LIFEWORK.

	Minimum	25th Percentile	Median	Mean	75th percentile	Maximum
NO_2 (µg/m^3)	8.87	19.07	22.92	23.59	27.34	88.72
$PM_{2.5}$ (µg/m^3)	14.86	16.17	16.56	16.58	17.04	21.33
$PM_{2.5}$ absorbance (10^{-5} m^{-1})	0.85	1.12	1.23	1.26	1.37	3.16
PM_{10} (µg/m^3)	23.73	23.96	24.44	24.77	25.15	34.54
Oxidative Potential (nmol DTT/min/m^3)	0.48	1.04	1.19	1.17	1.31	2.09

eTable 2. Odds Ratios of overall mortality per interquartile range width increase in mean air pollution exposure, evaluated with univariable and multivariable logistic regression models.

	Univariable models with minimal adjustment[a]		Multivariable model with full adjustment[b]		
Constituent	OR	95%CI	OR	95%CI	VIF
NO_2	0.98	(0.90;1.07)	0.93	(0.77;1.13)	5.30
$PM_{2.5}$	1.04	(0.95;1.12)	1.12	(0.95;1.32)	4.04
$PM_{2.5}$ absorbance	1.00	(0.93;1.07)	0.80	(0.59;1.08)	18.59
PM_{10}	1.01	(0.95;1.08)	1.17	(0.99;1.38)	7.21
Oxidative Potential	1.03	(0.94;1.12)	1.04	(0.93;1.17)	1.85

[a] Age, sex, BMI, smoking, CVD diagnosis.

[b] Age, sex, BMI, smoking, CVD diagnosis, COPD diagnosis, cancer diagnosis, education, income, normalized difference vegetation index.

BMI=body mass index; CVD=cardiovascular disease; COPD=chronic obstructive pulmonary disease; OR=odds ratio; CI=confidence interval; VIF=variance inflation factor.

eTable 3. Odds Ratios of overall mortality per interquartile range width increase in mean air pollution exposure, evaluated with a multivariable logistic regression model using multiple imputation by chained equations for missing values in the exposures.

	Multivariable model with minimal adjustment[a]		
Constituent	OR	95%CI	VIF
NO_2	0.98	(0.82;1.17)	5.11
$PM_{2.5}$	1.17	(0.99;1.37)	4.03
$PM_{2.5}$ absorbance	0.74	(0.55;0.98)	18.61
PM_{10}	1.21	(1.04;1.42)	7.23
Oxidative Potential	1.07	(0.96;1.19)	1.58

[a]Age, sex, BMI, smoking, CVD diagnosis.

BMI=body mass index; CVD=cardiovascular disease; OR=odds ratio; CI=confidence interval; VIF=variance inflation factor.

eTable 4. Odds Ratios of overall mortality per interquartile range width increase in mean air pollution exposure in a subset of constituents, evaluated with a multivariable logistic regression model.

	Multivariable model with minimal adjustment[a]		
Constituent	OR	95%CI	VIF
NO_2	0.91	(0.79;1.04)	2.85
$PM_{2.5}$	1.03	(0.94;1.14)	1.37
PM_{10}	1.06	(0.95;1.17)	3.10

[a]Age, sex, BMI, smoking, CVD diagnosis.

BMI=body mass index; CVD=cardiovascular disease; OR=odds ratio; CI=confidence interval; VIF=variance inflation factor.

eTable 5. Hazard Ratios of overall mortality per interquartile range width increase in mean air pollution exposure, evaluated with a Cox proportional hazards regression model.

	Cox model with minimal adjustment[a]	
Constituent	HR	95%CI
NO_2	1.00	(0.83;1.20)
$PM_{2.5}$	1.19	(1.02;1.39)
$PM_{2.5}$ absorbance	0.73	(0.55;0.96)
PM_{10}	1.22	(1.05;1.43)
Oxidative Potential	1.07	(0.97;1.19)

[a]Age, sex, BMI, smoking, CVD diagnosis.

BMI=body mass index; CVD=cardiovascular disease; HR=hazard ratio; CI=confidence interval.

eTable 6. Odds Ratios of overall mortality per interquartile range width increase in mean air pollution exposure, evaluated with univariate and multivariate generalized propensity score models[a] with varying trimming thresholds.

	GPS		mvGPS	
Constituent	OR	95%CI	OR	95%CI
Trimming=0.97				
NO_2	1.12	(1.03;1.21)	1.16	(0.99;1.36)
$PM_{2.5}$	1.13	(1.04;1.21)	1.08	(0.99;1.19)
PM_{10}	1.10	(1.04;1.17)	1.01	(0.90;1.14)
Oxidative Potential	1.10	(1.01;1.20)	1.01	(0.92;1.11)
Trimming=0.95				
NO_2	1.13	(1.04;1.22)	1.16	(0.99;1.36)
$PM_{2.5}$	1.13	(1.05;1.22)	1.08	(0.98;1.18)
PM_{10}	1.11	(1.05;1.18)	1.00	(0.88;1.13)
Oxidative Potential	1.11	(1.02;1.21)	1.00	(0.90;1.10)

[a] PS based on Age, sex, BMI, smoking, CVD diagnosis.

BMI=body mass index; CVD=cardiovascular disease; OR=odds ratio; CI=confidence interval; GPS=generalized propensity score; mvGPS=multivariate generalized propensity score.

eFigure 1. Relevance of 2-way interactions (H-statistics) in the overall mixture effect in predicting overall mortality, estimated with boosted regression tree.

	$PM_{2.5}$	$PM_{2.5}$absorbance	PM_{10}	Oxidative Potential
NO_2	0.18	0.33	0.03	0.21
$PM_{2.5}$		0	0.2	0.26
$PM_{2.5}$absorbance			0	0
PM_{10}				0

eFigure 2. Relative importance of mixture components in the overall effect of air pollution on overall mortality, estimated with weighted quantile sum.

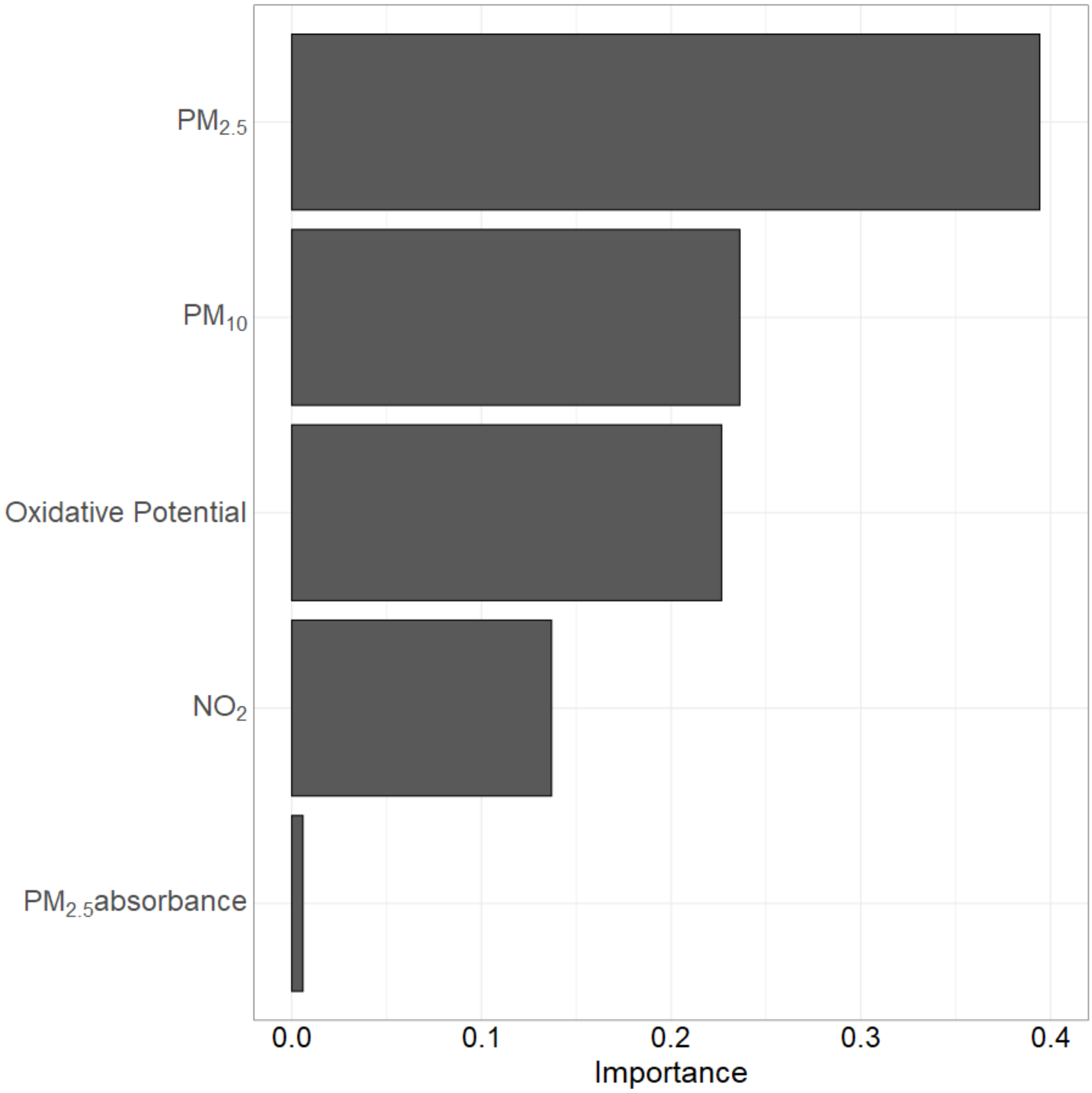

eFigure 3. Association between the mixture and overall mortality, estimated with weighted quantile sum.

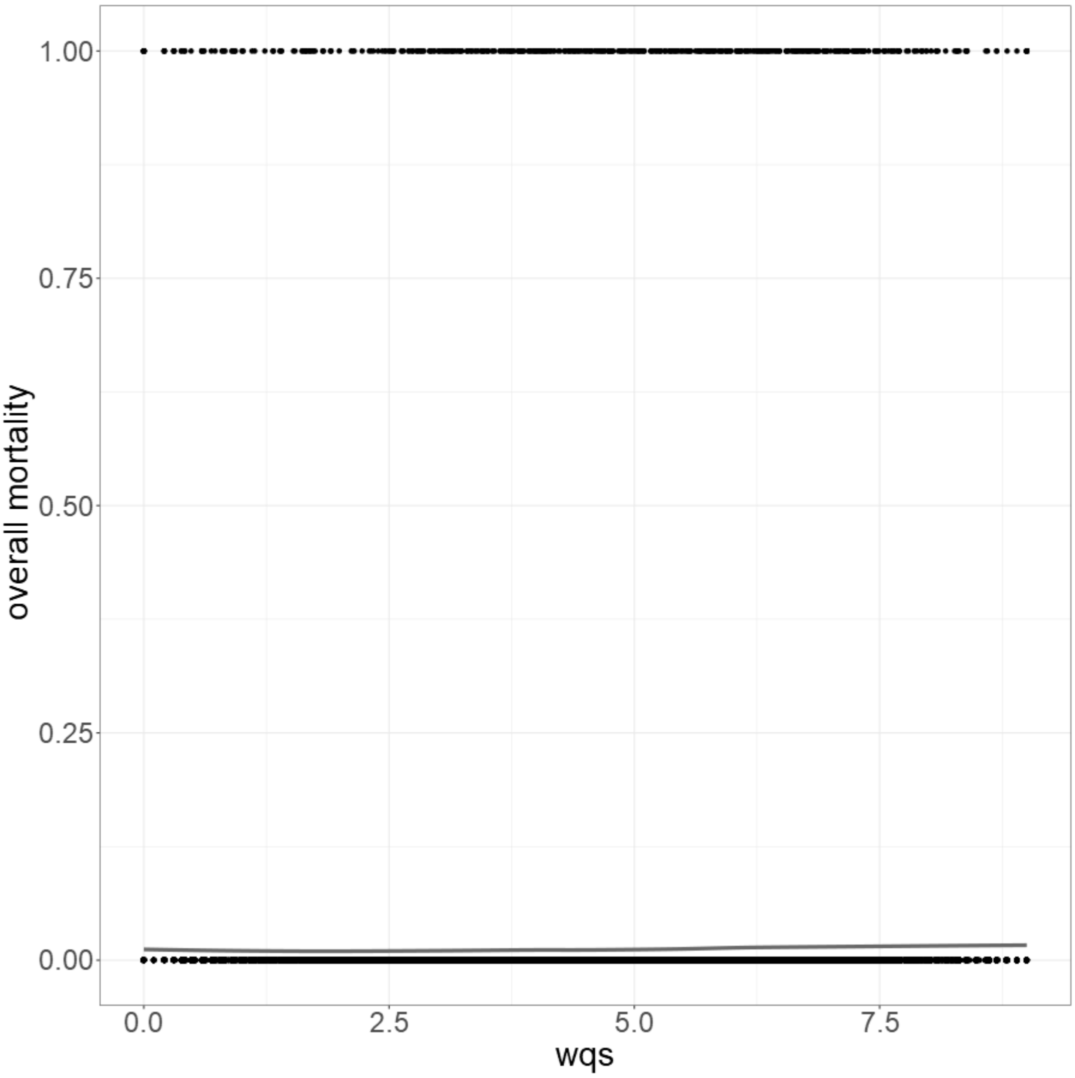

Supplement: Supplementary file 1 [file ede-33-514-s001.pdf]
